# Physical, chemical, and sensory properties of biscuits prepared from flour blends of unripe cooking banana, pigeon pea, and sweet potato

**DOI:** 10.1002/fsn3.590

**Published:** 2018-01-30

**Authors:** Abiodun A. Adeola, Ehimen R. Ohizua

**Affiliations:** ^1^ Food and Nutrition Research Programme Institute of Food Security, Environmental Resources and Agricultural Research Federal University of Agriculture Abeokuta Nigeria; ^2^ Department of Food Science and Technology Federal University of Agriculture Abeokuta Nigeria

**Keywords:** biscuit, cooking banana, nutrient composition, pigeon pea, sweet potato

## Abstract

Biscuits were produced from 14 flour blends of cooking banana (UBF), pigeon pea (PPF), and sweet potato (SPF). The physical properties, nutrient composition, and sensory characteristics of the biscuits were evaluated using standard methods. Data obtained were subjected to analysis of variance, and mean values were separated using Duncan's multiple range test. The hardness of the biscuit samples decreased as PPF increased, while the fracturability decreased with increase in UBF. Biscuits were significantly (*p* < .05) different in their nutrient composition, with the crude protein, crude fiber, ash contents, and dietary fiber content increasing as the PPF level increased. Cookies were rich in magnesium (576.54–735.06 mg/100 g) with favorable Na/K ratio (<1.0). The antinutritional factors in the biscuit samples were within permissible levels. Biscuits prepared from flour blend of 21.67% unripe cooking banana, 21.67% pigeon pea, and 56.67% sweet potato were the most preferred in terms of shape, mouthfeel, taste, crunchiness, and overall acceptability. Flour blends of unripe cooking banana, pigeon pea, and sweet potato could therefore be used as raw materials for the production of biscuits, with high protein, total dietary, and energy content.

## 1 INTRODUCTION

Biscuits are prominent ready‐to‐eat baked snack among the people, globally. The association of wheat consumption with such health problems as celiac disease makes it pertinent to utilize composite flour in biscuit manufacture (Kiin‐Kabari & Giami, 2015). Composite flour is desirable in this regard because it improves the nutritional value of food products such as bakery products, especially when blended with legumes such as pigeon pea (Preedy, Watso, & Patel, 2011). In fact, biscuits have been suggested as better use for composite flour than bread due to their ready‐to‐eat form, wide consumption, relatively long shelf life, and good eating quality (Bala, Gul, & Riar, 2015; Noorfarahzihah, Lee, Sharifudin, Mohd‐Fadzelly, & Hasmadi, 2014).

The use of banana flour in biscuits is desirable due to its contents of indigestible carbohydrate, mineral, and antioxidant capacity (Ovando‐Martinez, Sáyago‐Ayerdi, Agama‐Acevedo, Goñi, & Bello‐Pérez, 2009; Preedy et al., 2011). Pigeon pea, though grows well in Nigeria and is a good source of protein, is underutilized due to its hard‐to‐cook phenomenon (Fasoyiro & Arowora, 2013). The use of pigeon pea flour in the production of biscuits has been reported (Chinma et al., 2011; Okpala & Okoli, 2011; Silky & Tiwari, 2014). Sweet potato flour is used in baking as a dough conditioner and as an alternative flour in the production of gluten‐free products. It has been reported to improve the nutrient composition and toasting properties, reduce staling, and provide a distinctive flavor to several baked products such as bread and biscuits (Adeyeye & Akingbala, 2015; Noorfarahzihah et al., 2014; Onabanjo & Ighere, 2014).

This study which is a continuation of our earlier work (Ohizua et al., 2017) therefore evaluated some quality attributes of biscuits from composite flour of unripe cooking banana, pigeon pea, and sweet potato.

## 2 MATERIALS AND METHODS

### 2.1 Source of raw materials

Mature unripe cooking banana (*Musa cardaba* AAB) as earlier described by Ohizua et al. (2017), cream flesh sweet potato root (*Ipomea batatas*), and pigeon pea (*Cajanus cajan*) were obtained from local markets in Ibadan, Abeokuta, and Oshodi, respectively.

### 2.2 Preparation of flours from unripe cooking banana, pigeon pea, and sweet potato

Unripe cooking banana flour (UBF), pigeon pea flour (PPF), and sweet potato flour (SPF) were prepared according to the methods described by Ohizua et al. (2017). Banana fruits were washed, peeled under water treated with 0.05% (w/v) sodium metabisulphite, sliced to an average thickness of 1 mm, dried at 50°C for 24 hr in a Genlab Cabinet dryer (Model DC 500, Serial number 12B154), and milled using Fritsch hammer mill (Serial number: 15.302/982) equipped with a 250‐μm mesh sieve. Pigeon pea seeds were subjected to cleaning, boiling (20 min), dehulling, drying (60°C for 48 hr), cooling at room temperature, and milling using Fritsch hammer mill (Serial number: 15.302/982) equipped with a 250‐μm mesh sieve. Sweet potato roots were cleaned, cut into chips, soaked in 0.05% (w/v) sodium metabisulphite for 10–20 min to prevent browning, drained, dried in at 60°C for 48 hr, and milled in a Fritsch hammer mill (Serial number: 15.302/982) equipped with a 250‐μm mesh sieve.

### 2.3 Blending of flour

The method described by Ohizua et al. (2017) was used.

### 2.4 Preparation of biscuit

The modified recipe of Onabanjo and Ighere (2014) adopted after preliminary experimentation was as follows: flour (250 g), fat (63 g), sugar (63 g), salt (1 g), whole egg (20 ml), powdered milk (5 g), nutmeg (1.5 g), baking powder (1 g), and water (20–60 ml).

Biscuit was prepared using the traditional creaming method described by Chinma et al. (2011).The fat and sugar were mixed in a Kenwood mixer (HM 430) until the mixture was fluffy. Eggs and milk were added, while mixing continued. Baking powder, ground nutmeg, composite flour, and salt were introduced into the mixture to form a soft dough. The dough was removed from the bowl and kneaded on a flat surface to obtain a uniform mix. The kneaded dough was rolled out into sheets using a rolling pin and cut into the desired shape using a cutter. The cut mass was transferred to a greased baking tray. Baking was carried out at 180°C for 17 min. Biscuit made from 100% wheat flour served as the control sample.

### 2.5 Determination of physical properties of biscuit

The method described by Bala et al. (2015) was used, with slight modification, to evaluate the biscuit for the following parameters:

- Thickness of biscuits was determined by measuring the diameter of four biscuit samples placed edge to edge with a digital vernier caliper. An average of six values was taken for each set of samples. Average value for thickness was reported in millimeter.
- Diameter of biscuits was determined by placing four biscuit samples edge to edge and measuring with a digital vernier caliper. An average of six values was taken for each set of samples. Average value for diameter was reported in millimeter.
- Weight of biscuits was measured as average values of six individual biscuits with the help of an analytical weighing balance. Average value for weight was reported in grams.
- Spread ratio was calculated by dividing diameter by thickness.
- Texture profile analysis of biscuit samples: Hardness and fracturability analysis of the biscuit samples were determined using the TA‐XT Plus texture analyzer (Stable Micro Systems Serial No. 5014 England) according to the method described by Ahmed and Hussein (2014). The analyzer was set to perform single‐cycle measurements which were used for the determination of the first bite force of the product. The measurement speed of 2 mm/s and a distance of 5 mm were applied. The force–time plots were analyzed for hardness or breaking force (g) and fracturability (mm) to reach the peak. Textural attributes were measured in six independent samples.

### 2.6 Determination of color attribute of biscuit

The color intensity of the top and down (the one in contact with the baking tray) surfaces of biscuit samples was measured using a Konica Minolta Colour Measuring System (Chroma Meter CR‐410, Minolta LTD Japan). The lightness (L*), redness (a*), and yellowness (b*) values were obtained after calibrating the instrument using a white tile. Six replicate readings were taken for each biscuit formulation sample and the average value reported. The results were expressed in accordance with the CIE LAB system where:

L * is known as the lightness [L = 0 (black), L = 100 (white)],

a* (−a = greenness, +a = redness)

b * (−b* values = blueness, +b* value = yellowness)

### 2.7 Determination of chemical properties of biscuit

The proximate composition of the biscuit sample was determined in triplicate using the standard procedures of Association of Official Analytical Chemists (AOAC, 2010).

Total dietary fiber content was evaluated using the method of AOAC (2010).

The total carotenoid content of biscuit samples was determined by the method described by Ohizua et al. (2017). Briefly, the method involved filtering a mixture of biscuit sample and 70% methanol and washing the residue with acetone–petroleum ether. The extract was mixed with a solution of 10% KOH in methanol (v/v) in a separating funnel, which was allowed to stand for about 1 hr. In addition of petroleum ether and solution of sodium chloride to separating funnel, the lower layer was discarded and the upper layer treated with distilled water and anhydrous sodium sulfate. The filtrate was made up to 250 ml and the absorbance measured at 450 nm with a UV/Visible spectrophotometer (Model: CE 2021 2000 series, serial no 923‐41).

Calculation:

$$\text{Total carotenoids content}\left(\mu g/100g\right)=\frac{A\;\times \mathrm{Volume}\;\left(\mathrm{ml}\right)\;\times 10^{4}}{A_{1\;\mathrm{cm}}^{1\%}\;\times \mathrm{Weight}\;\mathrm{of}\;\mathrm{sample}}\pi r^{2}$$

where: *A* =Absorbance

Volume = total volume of extract

$A_{1\;\mathrm{cm}}^{1\%}$= Absorption coefficient of β‐carotene in petroleum ether (2,592).

The method described by Ohizua et al. (2017) was used to determine the mineral composition of biscuit. Sodium, potassium, and calcium were determined using the Jenway digital flame photometer, while the atomic absorption spectrophotometer (Thermo scientific S series Model GE Model No. 712354) was used to determine magnesium, iron, and manganese.

The method described by Ohizua et al. (2017) was used to determine the vitamin C content of the biscuit samples. Aqueous solution of ground biscuit sample was titrated with indophenol standard and the vitamin C calculated.

Calculation:

$$\text{Vitamin C content}\;\left(\mu g/100g\right)=\frac{0.0000833\times \mathrm{titre}\;\mathrm{value}\times 100000}{\mathrm{Weight}\;\mathrm{of}\;\mathrm{sample}\;(g)\times 20}$$

Tannin, oxalate, phytate, and trypsin inhibitor were determined as earlier described (Ohizua et al., 2017).

The energy content was estimated by calculation from fat, carbohydrate, and protein contents, using the Atwater's conversion factor: 4.0 kcal/g for protein, 9.0 kcal/g for fat, and 4.0 kcal/g for carbohydrate content.

### 2.8 Sensory evaluation of biscuit

Both the descriptive and discriminatory evaluation methods were used (Iwe, 2002). The organoleptic characteristics of the biscuit samples chosen by 15 trained judges were appearance (symmetry), color, aroma, mouthfeel, sweetness, hardness, crunchiness, and overall acceptability. Thereafter, a 50‐member panel evaluated coded biscuit samples based on the identified attributes using a 9‐point hedonic scale.

### 2.9 Data analysis

Data were analyzed using SPSS 21.0 software. Means with significant difference were separated by applying Duncan's multiple range test at 95% confidence level.

## 3 RESULTS AND DISCUSSION

### 3.1 Physical properties of biscuits

There were significant (*p* < .05) differences in the thickness, diameter, height, spread ratio, weight, hardness, and fracturability of the biscuit samples (Table 1). The thickness of the biscuits increased as the inclusion of SPF and UBF increased in the biscuit formulation. Values for height increased as the level of SPF increased, while the weight of the biscuit increased as level of PPF in the formulation increased. This may be due to the increasing protein content arising from incorporation of PPF. Spread ratio or diameter is used to determine the quality of flour used in preparing biscuits and the ability of the biscuit to rise (Bala et al., 2015). The higher the spread ratio of biscuit the more desirable it is (Chauhan, Saxena, & Singh, 2016). Hence, biscuit prepared from the flour blend containing 45% UBF, 10% PPF, and 45% SPF may be the most preferred based on spread ratio. This assertion is confirmed in Table 4 where there were no significant (*p* < .05) differences in the sensory attributes of this biscuit sample and the one adjudged to be the most acceptable. Biscuit sample prepared from 21.6:21.67:56.67 (UBF:PPF:SPF) flour blend had the highest overall acceptability. Similar findings with respect to the weight, diameter, and spread ratio were reported by other researchers (Mridula, Goyal, Bhargar, & Manikantan, 2007; Oluwamukomi, Oluwalana, & Akinbowale, 2011). Textural quality [hardness and fracturability] is a very important and desirable quality attribute for biscuit. Hardness, which is the peak force required to break the biscuit, decreased as the percentage inclusion of PPF in the formulation reduced. Fracturability measures the ability of a product to fight to regain its original status or form. Fracturability of the biscuit samples significantly (*p* < .05) reduced as the level of UBF increased. Apart from the baking conditions, the type, quantity of ingredients, and protein content of the flour used have been reported to influence its hardness and other textural attributes (Gaines, 1993; Pyler, 1982). The result suggests that blends having higher PPF and SPF levels would be suitable to maintain its shape during transportation and would fracture easily when chewed in the mouth name (Manley, 2001). Similar results for hardness of biscuit with increased incorporation of pigeon pea flour were reported by Silky and Tiwari (2014).

**Table 1:** Physical properties of biscuits prepared from flour blends of unripe cooking banana, pigeon pea, and sweet potato

UBF:PPF:SPF blend ratio	Thickness (mm)	Diameter (mm)	Height (mm)	Spread ratio	Weight (g)	Hardness (g)	Fracturability (mm)
10:80:10	6.11^ab^	38.46^ab^	37.83^abcd^	5.74^b^	7.49^e^	2,456.72^f^	3.34^a^
45:45:10	6.63^ab^	38.74^abcd^	37.92^bcd^	5.85^b^	5.35^abcd^	2,316.94^e^	3.72^b^
10:10:80	8.20^d^	39.30^d^	38.23^d^	4.79^a^	5.42^abcd^	3,285.04^m^	4.71^j^
21.67:56.67:21.67	6.43^ab^	39.09^bcd^	38.12^cd^	6.08^bc^	5.93^bcd^	2,771.33 ^l^	4.41^g^
45:10:45	6.05^a^	38.82^abc^	38.26^d^	6.48^c^	4.66^a^	3,092.47^k^	4.59^i^
10:45:45	6.64^ab^	38.48^ab^	37.42^abc^	5.80^b^	6.14^d^	2,945.73^j^	3.84^c^
10:10:80	7.96^cd^	39.24^cd^	38.23^d^	4.92^a^	5.44^abcd^	3,391.73^n^	4.148^d^
33.33:33.33:33.33	6.48^ab^	38.93^bcd^	38.43^d^	6.00^bc^	6.06^cd^	2,749.59^h^	4.72^k^
56.67:21.67:21.67	7.45^c^	38.40^ab^	37.31^ab^	5.16^a^	5.92^bcd^	2,164.51^d^	3.84^c^
10:80:10	6.72^b^	38.55^abc^	37.86^abcd^	5.77^b^	7.49^e^	2,550.59^g^	3.35^a^
80:10:10	6.13^ab^	38.16^a^	37.15^a^	6.26^bc^	5.17^abc^	1,632.03^a^	4.51^h^
45:10:45	6.05^a^	38.81^abcd^	38.26^d^	6.48^c^	4.66^a^	3,095.34^k^	4.40^f^
21.67:21.67:56.67	6.44^ab^	38.48^ab^	38.36^d^	5.98^bc^	5.05^ab^	3,817.64^n^	4.88 ^l^
80:10:10	6.16^ab^	38.16^a^	37.15^a^	6.24^bc^	5.20^abc^	1,759.61^b^	4.31^e^

Mean values with different superscripts within a column are significantly different (*p* < .05); UBF, unripe cooking banana flour; PPF, pigeon pea flour; SPF, sweet potato flour.

### 3.2 Color attribute of biscuit

Color is an important attribute because it can arouse individual's appetite. It is one of the parameters used for process control during baking and roasting, because brown pigments appear as browning and caramelization reactions progress (Pereira, Correia, & Guine, 2013). The L*, a*, and b* values of the biscuit samples were lower for the topside than downside (Table 2). The positive values of a* and b* values indicated the predominance of redness and yellowness in the biscuit samples. The color of the biscuits changed to dark brown from creamy yellow as the inclusion level of PPF and SPF increased. This may be due to the ingredient composition, air velocity in the oven, and red pigmentation resulting from the Maillard reaction or nonenzymatic browning which depends on the content of reducing sugars and amino acids or proteins on the surface, baking temperature, and time (Pereira et al., 2013). The current result is similar to the findings of other workers (Pereira et al., 2013).

**Table 2:** Color attributes of biscuit prepared from flour blends of unripe cooking banana, pigeon pea, and sweet potato

UBF:PPF:SPF blend ratio	Color of biscuit (topside)			Color of biscuit (downside)		
	L*	a*	b*	L*	a*	b*
10:80:10	27.60^cde^	16.10^f^	32.68^e^	54.58^ab^	16.70^cd^	41.29^i^
45:45:10	22.06^abc^	13.94^e^	22.82^b^	56.86^bc^	15.53^bc^	36.45^c^
10:10:80	35.76^f^	12.56^de^	30.01^de^	74.23^d^	10.57^a^	42.38^k^
21.67:56.67:21.67	34.59^ef^	14.28^e^	29.86^de^	50.55^a^	19.57^f^	37.36^d^
45:10:45	23.29^abc^	11.14^cd^	24.12^bc^	61.58^c^	14.68^b^	37.60^f^
10:45:45	34.76^f^	13.06^e^	30.24^de^	58.45^bc^	18.17^ef^	42.58 ^l^
10:10:80	34.60^ef^	13.23^e^	30.51^de^	74.06^d^	10.24^a^	42.38^k^
33.33:33.33:33.33	30.83^def^	12.71^de^	25.19^bc^	57.16^bc^	17.83^def^	40.30^h^
56.67:21.67:21.67	21.80^abc^	10.28^bc^	22.13^b^	53.87^ab^	16.82^cd^	35.51^b^
10:80:10	28.10^cde^	15.93^f^	32.71^e^	54.42^ab^	17.03^cd^	41.79^j^
80:10:10	19.77^ab^	8.74^ab^	17.53^a^	58.04^bc^	15.10^bc^	33.42^a^
45:10:45	23.79^bcd^	11.12^cd^	24.22^bc^	61.50^c^	14.51^b^	37.43^e^
21.67:21.67:56.67	35.73^ef^	13.17^e^	27.22^cd^	62.29^c^	16.00^bc^	40.03^g^
80:10:10	16.44^a^	8.57^a^	16.36^a^	58.04^bc^	15.10^bc^	63.11^m^

Means with different superscripts within a column are significantly different (*p* < .05); UBF, unripe cooking banana flour; PPF, pigeon pea flour; SPF, sweet potato flour.

### 3.3 Chemical properties of biscuit

There was a significant difference (*p* < .05) in the proximate composition, energy content, total carotenoid, and vitamin C content of the biscuits (Table 3). The moisture content of the biscuit samples increased as the inclusion levels of SPF and PPF increased. This may be attributed to the high water‐binding capacity of both flours which retained higher moisture content in the ultimate product (Anuonye, Jigam, & Ndaceko, 2012). The protein content increased as PPF increased but decreased as UBF and SPF increased. Similar observations were observed by other authors (Anuonye et al., 2012; Fasoyiro & Arowora, 2013; Silky & Tiwari, 2014; Tiwari, Brennan, Jaganmohan, Surabi, & Alagusundaram, 2011). Foods rich in protein content are of great nutritional importance in developing countries such as Nigeria where there is a prevalence of protein malnutrition (Anuonye et al., 2012; Okpala & Okoli, 2011). Protein is required by children for growth, repair, and maintenance of the body. It also acts as enzymes and hormones and maintains fluid, electrolyte and acid–base balance, and strong immune system (Mahan & Escott‐Stump, 2008). Proteins act as carriers for other nutrients such as lipids, vitamin A, iron, sodium, and potassium (Mahan & Escott‐Stump, 2008).

**Table 3:** Chemical properties of biscuits produced from flour blends of unripe cooking banana, pigeon pea, and sweet potato

Chemical attributes	UBF:PPF:SPF blend ratio													
	10:80:10	45:45:10	10:10:80	21.67:56.67:21.67	45:10:45	10:45:45	10:10:80	33.3:33.3:33.3	56.67:21.67:21.67	10:80:10	80:10:10	45:10:45	21.67:21.67:56.67	80:10:10
Moisture (g/100 g)	7.90^j^	7.00^f^	7.50^i^	5.90^a^	6.50^d^	6.60^e^	7.45^h^	7.10^g^	6.40^c^	7.90^j^	6.30^b^	6.50^d^	7.90^j^	6.30^b^
Crude protein (g/100 g)	16.86^j^	11.52^g^	6.10^a^	13.68^h^	6.34^b^	7.82^d^	6.11^a^	9.09^f^	8.15^e^	16.82^i^	6.87^c^	6.34^b^	7.82^d^	6.89^c^
Crude fat (g/100 g)	17.02^h^	16.65^e^	18.09^i^	18.84^j^	16.14^b^	16.19^c^	18.08^i^	16.71^f^	18.07^i^	16.96^g^	16.26^d^	16.14^b^	16.03^a^	16.25^d^
Crude fiber (g/100 g)	0.98^b^	3.02^h^	3.51^k^	2.15^f^	3.38^i^	1.93^d^	3.49^j^	2.05^e^	2.46^g^	0.98^b^	0.87^a^	3.38^i^	1.81^c^	0.87^a^
Total ash (g/100 g)	2.40^j^	0.90^a^	2.33^h^	1.88^d^	2.26^g^	1.78^c^	2.33^h^	1.77^c^	1.74^b^	2.39^i^	2.02^e^	2.26^g^	2.20^f^	2.02^e^
Carbohydrate (g/100 g)	54.84^a^	60.90^d^	62.46^e^	57.56^c^	65.37^j^	65.68^k^	62.53^f^	63.28^h^	63.18^g^	54.94^b^	67.69^m^	65.37^j^	64.24^i^	67.67 ^l^
Total dietary fiber (%)	19.05i	16.33^g^	11.73^a^	15.50^f^	13.63^b^	16.69^h^	11.74^a^	15.44^e^	13.74^c^	19.07^i^	14.61^d^	13.62^b^	14.60^d^	14.60^d^
Total carotenoid (μg/100 g)	93.00^a^	130.00^c^	177.00^ef^	105.00^ab^	118.00^bc^	172.00^e^	178.00^ef^	195.00^f^	174.00^e^	93.00^a^	90.00^a^	107.00^bc^	151.00^d^	88.00^a^
Mg (mg/100 g)	670.22^f^	702.74^k^	576.54^a^	652.74^d^	635.49^c^	675.63^h^	589.68^b^	674.53^g^	714.31^m^	700.67^j^	710.52 ^l^	684.86^i^	657.22^e^	735.06^n^
Tannin (mg/100 g)	23.31^g^	11.55^a^	16.86^d^	16.07^c^	32.93^i^	39.02^j^	16.90^d^	14.48^b^	20.99^f^	23.41^g^	24.00^h^	40.19^j^	17.81^e^	24.43^h^
Oxalate (mg/100 g)	9.96^d^	5.15^bc^	3.92^ab^	2.73^a^	5.50^c^	9.91^d^	3.91^ab^	5.12^bc^	5.60^c^	9.96^d^	3.50^ab^	5.59^c^	9.50^d^	3.24^ab^
Phytate (mg/100 g)	1.50^a^	3.75^b^	1.95^c^	4.00^i^	2.20^e^	2.00^d^	2.25^f^	4.30^j^	1.80^b^	1.50^a^	4.50 ^l^	2.20^e^	2.95^g^	4.55^k^
Trypsin inhibitor	1.01^a^	17.00^f^	12.00^e^	22.00^g^	10.00^d^	2.50^b^	10.00^d^	22.22^g^	6.00^c^	1.00^a^	27.50^h^	10.00^d^	1.00^a^	27.50^h^
Energy (kcal/100 g)	439.97^i^	439.55^e^	437.07^c^	454.48^i^	432.12^a^	439.73^g^	437.31^d^	439.89^h^	447.98^k^	439.71^f^	444.53^j^	432.12^a^	432.52^b^	444.50^j^

Mean values with different superscripts within a row are significantly different (*p* < .05); UBF, unripe cooking banana flour; PPF, pigeon pea flour; SPF, sweet potato flour.

The fat contents of the biscuit samples were similar to those reported by other researchers (Giwa & Ikujenlola, 2010; Silky & Tiwari, 2014) who used composite flours for biscuit manufacture. The fat content of the biscuits did not appear to vary much as the same quantities of fat were added. The variations observed in the fat contents of the biscuit samples, despite the same quantity of fat used in the recipe, may be due to variations in their moisture contents. The fat content of the biscuits was within the standard value (15%–20%) for soft dough biscuits (Manley, 2001). Similar values (15.1%–18.1%) were reported by other workers (Asif‐Ul‐Alam, Islam, Hoque, & Monalis, 2014; Chinma et al., 2011; Eneche, 1999; Silky & Tiwari, 2014) for biscuits prepared from composite flours containing different proportions of UBF, PPF, SPF, millet, wheat, and tigernut.

Fiber is known to aid the digestive system of humans. It has been reported that UBF, PPF, and SPF are good sources of fiber (Daramola & Osanyinlusi, 2006; Tiwari et al., 2011), and this could justify the results obtained for the crude fiber and total dietary fiber contents of the biscuits. Similar observation was reported by Silky and Tiwari (2014) and Chinma et al. (2011). The high ash contents of the biscuit samples attest to the nutritional report that PPF and SPF are good sources of calcium, sodium, magnesium, potassium, and iron (Torres, Frias, Grantito, & Vidal‐Valverde, 2007). The carbohydrate contents decreased as the percentage of PPF increased but increased as the percentage inclusion of UBF and SPF increased. This observation may be due to the low amount of carbohydrate in PPF and the high sugar content in UBF and SPF (Ayo‐Omogie & Ogunsakin, 2013; Silky & Tiwari, 2014). The two flour samples (UBF and SPF) are considered as starchy foods. The carbohydrate content of the biscuit samples was within the range (54.13%–69.02%) reported by Chinma et al. (2011) and Tiwari et al. (2011).

Dietary fiber is the indigestible part of plant material that helps increase roughage and bulk of stool, maintains a healthy intestine, and decreases the time waste material spends in the gastrointestinal tract (AACC, 2000; Potter & Hotchkiss, 1995). The total dietary fiber content of the biscuits increased as percentage inclusion of UBF and PPF increased. According to Ovando‐Martinez et al. (2009), Trinidad, Mallillin, Loyola, Sagum, and Encabo (2010), and Srikaeo, Sukanya, and Sopade (2011), unripe banana and legumes such as cowpea and pigeon pea are good sources of dietary fiber (21–41 g/100 g sample) and can be used in the preparation of functional foods. The recommended daily intake of dietary fiber for a healthy adult is 20–25 g (Dhingra, Michael, Rajput, & Patil, 2012). This implies that all the composite flour blends are good sources of dietary fiber. Food products high in dietary fiber are known as low glycemic index foods and have been shown to reduce postprandial blood glucose and insulin response and improve blood glucose and insulin concentration in subjects with diabetes mellitus (Trinidad et al., 2010). The total carotenoids content of the biscuit increased as the percentage of SPF increased in the formulation. Carotenoids are important micronutrients for human health. They function as a precursor of vitamin A. Some beneficial effects include enhancement of the immune system and decreased risk of degenerative diseases such as cancer, cardiovascular disease, age‐related muscular degeneration, and cataract formation (Kimura & Rodriguez‐Amaya, 2004).

There was a significant difference (*p* < .05) in the mineral composition of the biscuit samples. However, only magnesium was of significant value in the biscuit samples. The sodium, iron, potassium, and calcium contents of the biscuits ranged, respectively, from 0.019–0.02, 0.25–0.54, 0.31–0.69, 0.06–0.13, and 0.01–0.03 mg/100 g. The biscuit samples had high content of magnesium as the flour blends are rich sources of magnesium (Anuonye et al., 2012; Inyang & Ekop, 2015; Ohizua et al., 2017). Magnesium is a cofactor in more than 300 enzyme systems that regulate diverse biochemical reactions in the body, including protein synthesis, muscle and nerve function, blood glucose control, and blood pressure regulation. Magnesium keeps bones strong and heart rhythm steady (Wardlaw & Kessel, 2002). It is worthy of note that the sodium content of biscuits increased as the level of SPF increased, while the potassium content increased as the percentage inclusion level of UBF and SPF increased. The vitamin C content of the biscuit samples ranged from 0.12 to 0.21 μg/100 g, maybe as a result of its low level in the flour blends and heat processing (Ahmed, Akter, & Eun, 2010; Ohizua et al., 2017).

Antinutrients are substances that reduce the nutritional values of food by reducing the bioavailability, digestibility, and utilization of nutrients. There was a significant difference (*p* < .05) in the tannin, oxalate, phytate, and trypsin inhibitor contents of the biscuit samples. Tannins are polyhydric phenols which are present in virtually all parts of plants and have been found to inhibit trypsin, chymotrypsin, amylase, and lipase activities (Inyang & Ekop, 2015). The low tannin content is desirable as tannin forms insoluble complexes with proteins to decrease the digestibility of proteins (Uzeochina, 2007). Tannins can also provoke an astringent reaction in the mouth and decrease palatability of food, cause damage to intestinal tract, and enhance carcinogenesis (Onwuka, 2005). The low concentration of oxalate obtained in the study may be beneficial to man as low concentration of oxalate has been linked to reduction in blood cholesterol (Kaushal, Kumar, & Sharma, 2012). Oxalate is also known to form complexes with most essential trace elements including calcium thereby making them unavailable for enzymatic activities and other metabolic activities (Onwuka, 2005). The consumption of large dose of oxalic acid causes gastroenteritis, shock, convulsion, and renal damage. The lethal dose of oxalate has been reported to be between 2 and 5 g/kg for man. The biscuit samples are therefore safe, from the standpoint of oxalate level (Eneobong, 2001). Phytate level, though low, increased as the percentage inclusion of SPF and PPF increased. Pigeon pea and sweet potato are rich in phytate (Tiwari et al., 2011). It is known that phytate also forms complexes with protein at low and high pH and causes indigestion of food and flatulence. However, the low quantity of phytate in the biscuit samples should enhance the bioavailability of minerals such as iron, magnesium, and calcium (Anuonye, Onu, Egwin, & Adeyemo, 2009). The recommended limit for phytate is 250–500 mg/100 g (Ekop, Obot, & Ikpatt, 2008). Okpala and Okoli (2011) reported low phytic acid (0.56 to 0.70 mg/100 g) and tannin (0.36 to 0.51 mg/100 g) levels for cookies produced from pigeon pea, cocoyam, and sorghum flour blends. The trypsin inhibitor level increased as the percentage inclusion of UBF and PPF increased. Pigeon pea and banana are rich in trypsin inhibitors (Tiwari et al., 2011). It was generally observed in this study that the antinutritional content of the products was low and within the tolerable levels.

Energy content is the amount of calorie available from food through oxidation, and it is a function of the total protein, fat, and carbohydrates present in the food. The energy content of the biscuit samples increased as the protein content decreased. This was in conformity with reported trends (Iwe, Van Zauilichem, Ngoddy, & Ariahu, 2001). Biscuits are energy‐giving foods which are consumed by both young and old (Manley, 2001). The energy content of the biscuit samples was within the range (397–457 kcal/g) reported by Chinma et al. (2011) and Adeyeye and Akingbala (2015).

### 3.4 Sensory properties of biscuit

The sensory attributes of the biscuit samples are presented in Table 4. The biscuit samples varied significantly (*p* < .05) in terms of appearance, color, aroma, mouthfeel, taste, hardness, crunchiness, and overall acceptability. The appearance and color of the biscuit were strongly influenced by the SPF level. Biscuit samples with higher level of SPF had high scores in terms of appearance and color. Mouthfeel and aroma scores were more influenced by the PPF levels than other flours. Taste is an important attribute in acceptance of food product. The taste of the samples increased with the increasing inclusion of SPF. This finding confirms the report by Preedy et al. (2011) that SPF adds natural sweetness, color, and flavor to processed foods. Among the biscuit samples, biscuit sample produced from 21.67:21.67:56.67 (UBF:PPF:SPF) flour blends had the highest mean score (6.56) for hardness, while the one from 80:10:10 (5.80) had the least mean scores. Biscuit prepared from 21.67:56.67:21.67 (UBF:PPF:SPF) flour blend was least preferred in terms of color, aroma, mouthfeel, taste, and overall acceptability, while the one prepared from 21.67:21.67:56.67 (UBF:PPF:SPF) flour blend was the most preferred in terms of shape, mouthfeel, taste, crunchiness, and overall acceptability.

**Table 4:** Hedonic scores for the sensory attributes of biscuits produced from flour blends of unripe cooking banana, pigeon pea, and sweet potato

UBF:PPF:SPF blend ratio	Symmetry (shape)	Color	Aroma	Mouthfeel	Sweetness	Hardness	Crunchiness	Overall acceptability
10:80:10	6.63^bcd^	6.82^d^	6.14^bc^	5.80^a^	6.06^ab^	5.80^a^	5.39^a^	6.41^bc^
45:45:10	6.24^bc^	6.32^bcd^	6.16^bc^	6.68^bcd^	6.32^bc^	6.32^abc^	6.24^bc^	6.52^bc^
10:10:80	6.96^cd^	6.68^cd^	6.44^cd^	6.24^abc^	5.92^ab^	6.44^abc^	6.56^cd^	6.52^bc^
21.67:56.67:21.67	5.28^a^	4.76^a^	5.32^a^	5.68^a^	5.40^a^	5.92^ab^	6.48^cd^	5.76^a^
45:10:45	6.48^bcd^	5.88^b^	6.44^cd^	6.08^abc^	6.20^abc^	6.64^cd^	6.68^cd^	6.68^bc^
10:45:45	6.44^bcd^	6.48^bcd^	5.92^abc^	6.08^abc^	6.00^ab^	5.80^a^	5.64^ab^	6.20^ab^
10:10:80	6.96^cd^	6.72^cd^	6.46^cd^	6.26^abcd^	5.90^ab^	6.46^abc^	6.56^cd^	6.52^bc^
33.33:33.33:33.33	6.60^bcd^	6.40^bcd^	6.52^cd^	6.40^abcd^	5.96^ab^	5.84^a^	6.20^bc^	6.48^bc^
56.67:21.67:21.67	6.12^b^	6.00^bc^	5.64^ab^	5.88^ab^	5.52^ab^	5.92^ab^	6.32^bc^	6.24^ab^
10:80:10	6.70^bcd^	6.88^d^	6.16^bc^	5.81^a^	6.08^ab^	5.86^a^	5.50^a^	6.52^bc^
80:10:10	6.38^bcd^	6.32^bcd^	6.22^bc^	6.14^abc^	5.94^ab^	6.32^abc^	6.52^cd^	6.58^bc^
45:10:45	6.48^bcd^	5.88^b^	6.44^cd^	6.08^abc^	6.20^abc^	6.64^cd^	6.68^cd^	6.68^bc^
21.67:21.67:56.67	7.08^d^	6.56^bcd^	6.48^cd^	6.72^cd^	6.92^cd^	6.56^abc^	6.72^cd^	7.04^c^
80:10:10	6.38^bcd^	6.32^bcd^	6.18^bc^	6.16^abc^	5.96^ab^	6.28^abc^	6.50^cd^	6.58^bc^

Mean values with different superscripts within a column are significantly different (*p* < .05); UBF, unripe cooking banana flour; PPF, pigeon pea flour; SPF, sweet potato flour.

## 4 CONCLUSION

The physical, chemical, and sensory characteristics of biscuit produced from flour blends of unripe banana, pigeon pea, and sweet potato were studied. The thickness of biscuit samples increased as the level of sweet potato flour and unripe cooking banana flour increased, while the hardness decreased with increase in pigeon pea flour. There was a predominance of redness and yellowness in the biscuit samples. The crude protein, crude fiber, and total ash contents of biscuits increased as the PPF level increased. The biscuit samples had high energy content and were rich in magnesium with favorable Na/K ratio (<1.0) and with antinutritional contents within the tolerable levels.

## CONFLICT OF INTERESTS

The authors declare that there is no conflict of interests.
